# The Relationship Between Perceived Discrimination, Acculturation Attitudes, and Adaptation among Anglophone African Immigrants in Russia: The Moderating Role of Neuroticism

**DOI:** 10.11621/pir.2023.0105

**Published:** 2023-03-30

**Authors:** Sepase K. Ivande, Tatiana Ryabichenko

**Affiliations:** a Motivation and Intercultural Relations Lab, Department of Psychology, University of Victoria, BC, Canada; b School of Psychology, HSE University, Moscow, Russia; c International Laboratory for Social Integration Research, HSE University, Moscow, Russia

**Keywords:** Perceived discrimination, neuroticism, acculturation attitudes, psychological adaptation, sociocultural adaptation, African immigrants, Russia

## Abstract

**Background:**

Perceived discrimination is an acculturative stressor that negatively predicts psychological and socio-cultural adaptation, partially mediated by the individual’s acculturation attitudes. However, despite being under similar conditions of high perceived discrimination, some African immigrants in Russia appear to adapt more successfully than others. Why the individual differences? Neuroticism is a trait that intensifies the experience of negative emotions and sensitivity to stress. Perhaps it amplifies the reaction to acculturative stressors (e.g., perceived discrimination) in terms of acculturation attitudes, with significant implications for adaptation.

**Objective:**

This study sought to determine whether the personality trait of neuroticism influences how African immigrants in Russia react to perceived discrimination in terms of their acculturation attitudes and how this may relate to adaptation.

**Design:**

A moderated mediation analysis was carried out, investigating neuroticism as a moderator in the relationship between perceived discrimination, acculturation attitudes, and adaptation of African immigrants in Russia (*N* = 157).

**Results:**

Perceived discrimination was found to be strongly associated with poor psychological and sociocultural adaptation, which was partially mediated by the integration attitude; neuroticism strengthened this indirect negative association.

**Conclusion:**

When highly neurotic African immigrants perceived elevated levels of discrimination, they were more averse to adopting a positive attitude toward integration, and as a result, were more maladapted. This result suggests that the differences in the levels of adaptation among African immigrants in Russia under similar conditions of high perceived discrimination may be partially due to their levels of neuroticism.

## Introduction

The past few decades have seen a significant increase in the African immigrant population in the Russian Federation (Bondarenko, 2017; Bondarenko, Googueva et al., 2009; Fidan & Aras, 2010; Simmons, 2014). The number of African immigrants in Russia is estimated to be more than 50,000 and is forecast to steadily rise over the coming years (Bondarenko, 2017; Gribanova, 2009). Despite the burgeoning population of African immigrants in Russia, there have only been a handful of studies on the acculturation of Africans in Russia (Boltovskaja, 2010; Bondarenko, 2017; Bondarenko et al., 2009; J. Allina-Pisano & E. Allina-Pisano, 2007; Simmons, 2014).

Bondarenko et al. (2009) interviewed some African immigrants on their life experiences as immigrants in Russia while studying their sociocultural adaptation. They found that most of the interviewees reported a high level of perceived discrimination and appeared to be relatively maladapted in Russian society. However, those who regarded themselves as “well-adapted” were more likely to consider native Russians’ attitudes toward them as positive or tolerant, meaning that they perceived less discrimination (Bondarenko, 2017). It is well established that one of the main acculturation conditions that impedes successful adaptation of immigrants, in general, is perceived discrimination from the host culture (Berry et al., 2006; Berry & Sabatier, 2010; Güler & Yıldırım, 2022; Hashemi et al., 2019; Jasinskaja-Lahti & Liebkind, 2001; Kulis et al., 2009; Noh & Kaspar, 2003; Robinson, 2005; Te Lindert et al., 2008). Perceived discrimination is not just detrimental to adaptation directly but also indirectly through acculturation attitudes, as it reduces the possibility of adopting a positive attitude toward integration, and thus impedes successful adaption. In this regard, perceived discrimination possibly influences the acculturation attitudes of African immigrants in Russia, which could explain their varying levels of adaptation.

Although the majority of the African immigrants Bondarenko and colleagues interviewed seemed maladapted in Russian society, a handful appeared well-adapted and integrated. Most of the well-adapted and integrated African immigrants were highly educated Soviet or Russian university alumni (Bondarenko, 2017; Bondarenko et al., 2009). However, there were cases of African immigrants who managed to successfully integrate and adapt to Russian society despite being under stressful acculturation conditions like perceived discrimination and lack of access to quality education (Simmons, 2014). This indicates the salience of other factors that might be influencing their psychological and sociocultural adaptation.

Research has identified several other factors that play a role in the acculturation process. These include perceived ethnic support, personality traits, etc. (Arends-Tóth & Van de Vijver, 2006; Hasan et al., 2021; Kvernmo & Heyerdahl, 2004; Van der Zee et al., 2016). Some of these are potential moderators in the relationship between perceived discrimination, acculturation attitudes, and adaptation. When evaluating variance among individuals in similar sociocultural circumstances, it is logical to consider dispositional characteristics like personality traits as possible causes.

Perceived discrimination is an acculturative stressor that elicits negative emotions (Berry, 2006; Britt-Spells et al., 2018). In this regard, neuroticism is particularly intriguing because it is the trait that determines sensitivity to stress and consequent negative emotions (Gunthert et al., 1999; McCrae & John, 1992; Steenhaut et al., 2018). Thus, individuals showing higher levels of neuroticism may be more vulnerable to the negative emotions elicited by perceived discrimination, potentially intensifying their reaction to discrimination. The issue thus arises: does neuroticism amplify the detrimental effects of perceived discrimination on adaptation? Previous research has revealed that certain components of neuroticism (*e.g.,* emotional reactivity, or an individual’s tendency to react to stressful events with negative affect) amplify the direct negative impact of perceived discrimination on psychological well-being (Stein et al., 2016).

However, there is still a gap in terms of how neuroticism may influence the indirect impact of perceived discrimination on well-being/adaptation, such as how neuroticism may interact with perceived discrimination to predict one’s acculturation attitude and its implication for adaptation. Thus, this study aimed to determine the moderating role of neuroticism in the indirect relationship between perceived discrimination and adaptation via the acculturation attitudes of African immigrants in Russia. The results could offer insight into how and why some African immigrants, under similar stressful acculturation conditions like high perceived discrimination, adapted better than others.

### The Conceptual Framework of Acculturation

Acculturation can be defined as “the dual process of cultural and psychological change that takes place as a result of contact between two or more cultural groups and their members” (Berry, 2005, p. 698). According to the framework established by Arends-Toth and van de Vijver (2006), acculturation variables can be divided into three groups: acculturation conditions, orientations, and outcomes. Acculturation conditions, or the background and context under which the acculturation process occurs (*e.g.,* perceived discrimination, personality traits, etc.). The variations in conditions can influence the immigrant’s acculturation orientation/attitudes (*i.e*., how the acculturating groups or individuals decide to become involved with and relate with each other).

Underlying the acculturation attitudes are the issues of maintaining one’s heritage/ethnic culture and/or adopting the mainstream culture. The intersections of these two issues result in four possible acculturation attitudes: 1) integration attitude (engage in both the heritage and mainstream cultures); 2) assimilation attitude (reject their heritage culture and adopt the mainstream culture); 3) separation attitude (maintain their heritage and reject the mainstream culture); and 4) marginalization attitude (reject both the heritage and mainstream cultures) (Berry, 2005). Which acculturation attitude is chosen can then influence the level of adaptation, which is typically assessed with two latent variables: psychological adaptation (feelings of well-being and satisfaction), and sociocultural adaptation (ability to fit in and negotiate interactive aspects of the new culture and one’s own culture) (Searle & Ward, 1990).

### Perceived Discrimination, Acculturation Attitudes, and Adaptation

Perceived discrimination is the subjective perception or experience of the salience of unfairness in an outcome attributed to prejudice (Schmitt & Branscombe, 2002). It is detrimental to psychological well-being in that it can lead to adverse psychological symptoms, such as low self-esteem, depression, aggression, insecurity, etc. (Britt-Spells et al., 2018; DeWall & Bushman, 2011; Model et al., 2009; Stein et al., 2016). Perceived discrimination can also influence adaptation indirectly through acculturation attitudes (Berry et al., 2006; Berry & Sabatier, 2010; Giuliani et al., 2018; Te Lindert et al., 2008).

Each of Berry’s four acculturation attitudes results in a different level of adaptation by the immigrants (Berry, 1997, 2003, 2005; Berry et al., 2006; Berry & Sabatier, 2010; Hashemi et al., 2019; Te Lindert et al., 2008; Ward & Rana-Deuba, 1999). Integration usually results in good sociocultural and psychological adaptation (Bierwiaczonek & Kunst, 2021), while marginalization usually results in poor psychological and sociocultural adaptation. Assimilation can facilitate sociocultural adaptation but may be detrimental to psychological adaptation, while separation can facilitate psychological adaptation but may impede sociocultural adaptation (Berry et al., 2006; Berry & Sabatier, 2010; Guerra, et al., 2019; Ward & Rana-Deuba, 1999). Perceived discrimination typically orients immigrants/ minorities away from participating in the mainstream culture, thus decreasing their likelihood of adopting an integrative or assimilationist attitude while increasing their tendency to adopt a separation or marginalization attitude (Berry et al., 2006; Berry & Sabatier, 2010; Guerra, et al., 2019; Hashemi et al., 2019; Te Lindert et al., 2008).

According to the rejection-identification model, individuals may increase their orientation towards strong ethnic identification under conditions of high perceived discrimination, which can help them maintain psychological well-being in the face of societal devaluation (Branscombe et al., 1999; Hashemi et al., 2019). This explains why perceived discrimination may positively relate to a separation attitude. In addition, social identity theory contends that people’s self-image is derived from the group with which they identify, so they are motivated to identify with a positively valued group to preserve a positive self-image (Tajfel & Turner, 1979). When a group’s value (esteem) is threatened (*e.g*., through discrimination), a person may attempt to dis-identify from his or her in-group to protect his or her self-esteem, and become increasingly averse to the source of the threat (*e.g*., the discriminatory mainstream culture for immigrants/minorities) (Tajfel & Turner, 1986). This explains why perceived discrimination may positively relate to the marginalization attitude.

In accordance with all these theories, what appears clear is that perceived discrimination severely decreases the likelihood that immigrants/ minorities will simultaneously engage in their heritage and the mainstream culture, implying a strong negative association with an integrative attitude. Considering that the integration attitude tends to positively predict both psychological and sociocultural adaptation (Bierwiaczonek & Kunst, 2021), a negative orientation toward integration is likely to result in maladaptation.

### Neuroticism as a Moderator in the Relationship between Perceived Discrimination, Acculturation Attitudes, and Adaptation

Neuroticism is characterized by a chronic level of emotional instability and proneness to psychological distress. People with high levels of neuroticism are often insecure in their self-image, anxious, and paranoid. They tend to over-think and exaggerate the significance of their problems, and dwell a lot on the negative aspects of things (McCrae & John, 1992). Neuroticism predicts psychological and sociocultural maladaptation (Bakker et al., 2005; Friedrich & Alvarez, 2020; Ryder et al., 2000; Van der Zee et al., 2016; Van der Zee & Van Oudenhoven, 2013, 2014; Ward et al., 2004). It also has been consistently found to positively predict marginalization attitudes (Friedrich & Alvarez, 2020; Ryder et al, 2000; Schmitz & Berry, 2011).

However, findings on the relationship between neuroticism and integration attitude have been mixed. While Schmitz and Berry (2011) found that neuroticism had a slightly negative but not significant relationship with integration attitudes among immigrants in Germany, Friedrich and Alvarez (2020) found that neuroticism had a slightly positive significant relationship with integration attitudes among Argentine immigrants in Israel. This variation in results suggests that the relationship could be contextual. Perhaps dependent on the level of perceived acculturative stress?

Generally, acculturation can be a stressful phenomenon (Berry, 2006). One of the main acculturative stressors is perceived discrimination. Perceived discrimination is a form of acculturative stress that elicits negative emotions. Given that neuroticism influences one’s susceptibility to stress and negative emotions (Gunthert et al., 1999; McCrae & John, 1992), we can expect neuroticism to have a potent interaction with perceived discrimination. Someone with a high level of neuroticism should be more affected by the negative emotions elicited by perceived discrimination, potentially amplifying their reaction to perceived discrimination.

As acknowledged earlier, perceived discrimination mostly orients immigrants or minorities away from participating in or adopting the mainstream culture. However, it doesn’t necessarily mean that they will increasingly opt to preserve their heritage culture (separation attitude). Sometimes, they increasingly orient away from both the mainstream and the heritage culture (marginalization attitude) under conditions of perceived discrimination (Berry et al., 2006). Neuroticism is especially relevant in this regard because individuals higher in neuroticism tend to have fragile self-esteem, and be more self-conscious (F. Amirazodi & M. Amirazodi, 2011; McCrae & John, 1992). In this regard, their neuroticism may increase their tendency to dis-identify with their in-group in order to protect their self-esteem under conditions of perceived discrimination. However, they also tend to be higher in rejection sensitivity (Brookings et al., 2003; Wilhelm et al., 2004) — *i.e.,* the tendency to readily perceive, anxiously expect, and intensely react to cues of rejection (*e.g.,* discrimination) (Downey & Feldman, 1996). Thus, their tendency to overreact to cues of rejection may also intensify their aversion to participating in the mainstream culture, which is the typical reaction to discrimination.

In light of this, we can hypothesize that neuroticism strengthens perceived discrimination’s indirect negative association with psychological and sociocultural adaptation, by strengthening perceived discrimination’s negative association with integration attitude, as well as its positive association with marginalization attitude.

### Current Study

**Figure 1:**
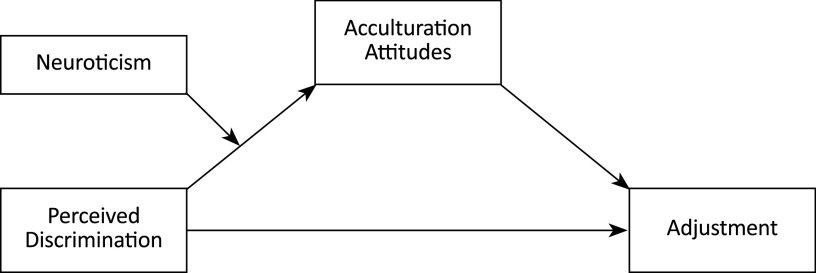
A moderated mediation model for examining the moderating role of neuroticism in the relationship between perceived discrimination, acculturation attitudes, and adaptation

This study aimed to determine the moderating role of neuroticism in the relationship between perceived discrimination, acculturation attitudes, and adaptation of African immigrants in Russia. *Figure 1* shows the theoretical model.

Within the framework of the model, this study tested the following hypotheses.

1. Perceived discrimination is directly associated with adaptation, as well as indirectly through the acculturation attitudes of African immigrants in Russia.
2. Neuroticism moderates the indirect relationship between perceived discrimination and adaptation by directly affecting the relationship between perceived discrimination and acculturation attitudes.

## Methods

### Participants

The sample consisted of 157 participants from five different Anglophone African countries: Nigeria (78.3%), Ghana (12.7%), Namibia (3.8%), Zambia (2.5%), and Kenya (2.5%). The participants were primarily male (69.4%). The mean age of the sample was 25.27 years (SD = 4.67), ranging from 18 years to 55 years. Approximately 60% of the participants had a bachelor’s degree; 26.8% a master’s degree; 7.6% a doctorate; and 5.7% only a secondary/high school education.

### Procedure

The participants were recruited by means of convenience sampling via an online group forum of Anglophone African immigrants in Russia. The members were urged to complete the survey in English. Participation was anonymous and voluntary.

### Materials

The responses to the survey questions were scored on a seven-point Likert scale, with answer options ranging from 1 (strongly disagree) to 7 (strongly agree). Confirmatory factor analyses were carried out to test the reliability and validity of the scales for use in the study sample. See the appendix for the list of items for all measures.

#### Perceived Discrimination

Four (4) items were used to measure perceived discrimination. They were adopted and adapted from the perceived discrimination scale developed by Phinney, Madden, and Santos (1998). Cronbach’s alpha was .88 for the study sample. The fit indices were acceptable for the study sample (χ^2^/df = 1.79; CFI = .99; RMSEA = .07, PCLOSE = .28).

#### Neuroticism

Five (5) items were adopted and adapted from the neuroticism sub-scale of the 120-item IPIP-NEO (International Personality Item Pool [IPIP], n.d.). The fit indices were acceptable for this study sample (χ^2^/df = 2.03; CFI = .97; RMSEA = .08, PCLOSE = .20), and the scale was fairly reliable (α = .70).

#### Acculturation Attitudes

We used the four-fold measurement method to assess acculturation attitudes. The instrument used contained sixteen (16) items adopted and adapted from the acculturation attitude scale developed by Kim (2010). The fit indices were acceptable for this study sample (χ^2^/df = 1.62; CFI = .89; RMSEA = .07, PCLOSE = .06). These items measure the four acculturation attitudes *— i.e*., Integration (*α* = .68), separation (*α* = .60), assimilation (*α* = .62), and marginalization (*α* = .80) — across four life domains (*i.e.,* friendship, society, cultural events, and lifestyle). Each attitude is measured with four (4) items, 1 for each life domain.

We discovered that the Cronbach’s alphas for the scales integration, separation, and assimilation were rather low. This is most likely because each item measures the participants’ cultural orientation in a different life domain. A participant may not adopt the same attitude in every life domain to the same degree. For example, a participant may not adopt separation in the friendship domain as much as they may in the cultural events domain. Thus, Cronbach’s alpha is unlikely to be high. The original scales developed by Kim (2010) also reported Cronbach’s alpha values in the range of 0.6–0.8 across multiple samples. Nevertheless, the rule of thumb is that 0.6 or above is acceptable for cross-sectional correlational studies (Ursachi et al., 2015). Given that all our scales crossed that threshold, we proceeded to use them for our analysis.

#### Adaptation

Eight (8) items were used to measure adaptation, adopted and adapted from the Brief Psychological and Sociocultural Adaptation Scale (BPSAS) (Demes & Geeraert, 2014). The fit indices were acceptable for this study sample (χ^2^/df = 1.88; CFI = .95; RMSEA = .09, PCLOSE = .03). The BPSAS is made up of two subscales, namely, the Brief Sociocultural Adaptation Scale (BSAS), and the Brief Psychological Adaptation Scale (BPAS). There were four (4) items from BSAS (*α* = .84), and four (4) items from the BPAS (*α* = .60).

### Data Analysis

For the data analysis, we used SPSS and AMOS. To test hypothesis 1, we performed mediation analysis using the SPSS add-on known as *Process-macro*, developed by Andrew Hayes (2017). We used model 4 in *Process-macro (i.e*., a mediation model). To test hypothesis 2, we conducted a moderated mediation analysis also using *Process-macro*. We used model 7 in *Process-macro* (*i.e.,* a moderated mediation model).

## Results

### Descriptive Statistics of Variables

*Table 1* shows the intercorrelations among all variables. *Table 2* shows the descriptive statistics for the variables and mean comparisons based on the sample’s socio-demographics.

**Table 1:** Correlations amongst variables (Spearman’s correlation)

	1	2	3	4	5	6	7
1. Perceived Discrimination	1						
2. Neuroticism	.19^*^	1					
3. Integration	–.27**	–.15	1				
4. Assimilation	–.06	.20*	.21**	1			
5. Separation	.21**	.08	–.32**	–.08	1		
6. Marginalization	.22**	.27**	–.41**	.09	.09	1	
7. Sociocultural Adaptation	–.35**	–.28**	.53**	.41**	–.22*	–.18*	1
8. Psychological Adaptation	–.60**	–.28**	.53**	.07	–.15	–.29**	.45**

Note. N = 157. **p< .01; *p< .05.

**Table 2:** Descriptive statistics of study variables, compared by socio-demographics

		PD	Neu	Intg	Assm	Sepr	Marg	SCA	PA
**Total Sample [M(SD)]**		3.9	3.7	4.7	3.1	3.9	3.5	4.7	5.1
		(1.6)	(1.3)	(1.3)	(1.1)	(1.1)	(1.6)	(1.4)	(1.0)
Gender	Male M(SD)	3.7	3.5	4.8	3.2	3.8	3.4	4.9	5.1
		(1.5)	(1.1)	(1.1)	(1.1)	(1.1)	(1.4)	(1.2)	(.97)
	Female [M(SD)]	4.2	4.2	4.5	3.0	4.1	3.8	4.1	4.9
		(1.5)	(1.5)	(1.4)	(1.1)	(1.1)	(1.7)	(1.3)	(1.2)
	*t* (155)	–1.7	–2.8**	1.4	1.0	–1.5	–1.4	3.8***	.65
Education	High School [M(SD)]	4.2	3.6	4.8	3.1	3.7	3.8	4.0	5.1
		(1.3)	(1.3)	(1.2)	(1.0)	(1.2)	(1.1)	(1.2)	(.40)
	Bachelors [M(SD)]	3.9	3.6	4.8	2.9	3.8	3.4	4.7	5.0
		(1.6)	(1.2)	(1.2)	(1.0)	(1.0)	(1.5)	(1.3)	(1.0)
	Masters [M(SD)]	3.8	3.9	4.4	3.4	3.8	3.7	4.7	5.1
		(1.7)	(1.4)	(1.3)	(1.1)	(1.2)	(1.5)	(1.5)	(1.2)
	Doctorate [M(SD)]	3.5	3.5	4.8	3.4	4.5	3.4	5.3	5.2
		(1.3)	(1.0)	(1.2)	(1.8)	(1.3)	(1.2)	(.86)	(.97)
	*F* (3, 153)	.48	1.1	1.8	2.6	1.2	1.1	1.3	.17
Time in Russia	< 2 yrs [M(SD)]	3.3	3.6	4.9	3.1	3.8	3.3	4.8	5.3
		(1.3)	(1.1)	(1.1)	(1.1)	(.94)	(1.6)	(1.3)	(.87)
	2–4 yrs [M(SD)]	4.0	3.8	4.6	.2.9	3.8	3.7	4.3	4.8
		(1.6)	(1.2)	(1.3)	(86)	(1.0)	(1.7)	(1.1)	(1.1)
	> 4 yrs [M(SD)]	4.0	3.7	4.7	3.3	3.9	3.5	4.8	5.0
		(1.6)	(1.3)	(1.2)	(1.2)	(1.2)	(1.5)	(1.4)	(1.1)
	*F* (2, 154)	1.9	.13	.49	1.1	.12	.46	1.2	1.6
Country of Origin	Nigeria [M(SD)]	3.9	3.7	4.7	3.1	3.9	3.5	4.7	5.0
		(1.6)	(1.2)	(1.2)	(1.1)	(1.1)	(1.5)	(1.3)	(1.0)
	Ghana [M(SD)]	3.7	3.9	4.6	3.1	4.0	2.9	5.1	5.1
		(1.7)	(1.3)	(1.5)	(1.0)	(1.2)	(1.4)	(1.4)	(1.0)
	Namibia [M(SD)]	4.2	3.4	5.0	2.4	4.0	2.1	4.1	5.6
		(1.6)	(.72)	(1.5)	(.80)	(1.2)	(1.2)	(.95)	(.61)
	Zambia [M(SD)]	4.6	4.9	4.6	3.4	3.7	3.3	3.9	4.9
		(1.3)	(1.2)	(1.7)	(.66)	(.72)	(2.4)	(.92)	(1.8)
	Kenya [M(SD)]	5.1	4.3	4.6	3.1	3.8	3.8	5.3	4.6
		(2.6)	(1.6)	(1.8)	(1.2)	(.99)	(1.3)	(1.5)	(1.3)
	*F* (4, 152)	.92	1.3	.09	.77	.19	1.8	1.4	.67

Note. N = 157. ***p< .001; **p< .01; *p< .05; PD = Perceived Discrimination; Neu = Neuroticism; Intg = Integration; Assm = Assimilation; Sepr = Separation; Marg = Marginalization; SCA = Socio-cultural Adaptation; PA = Psychological Adaptation.

In comparing genders through their t-test results, we found that the females (*M* = 4.2, *SD* = 1.5) were higher in neuroticism than the males (*M* = 3.5, *SD* = 1.1), which was significant: *t* (155) = −2.8, *p* <.01. Males (*M* = 4.9, *SD* = 1.2) were also more socio-culturally adapted than females (*M* = 4.1, *SD* = 1.3). The difference was also significant: *t* (155) = 3.8, *p* <.001.

There were no statistically significant differences on any variables, when compared by level of education, length of time spent in Russia, or country of origin (see *Table 2*).

### The Relationship Between Perceived Discrimination, Acculturation Attitudes, and Adaptation

Perceived discrimination had a significantly negative direct association with sociocultural (β = -.15, *p* < .01) and psychological adaptation (β = -.43, *p* < .01), after the indirect associations via the acculturation attitudes and other covariates (age, gender, education, length of time spent in Russia, and neuroticism) were accounted for (see *Table 3*).

**Table 3:** Multiple regression analysis predicting adaptation of African immigrants in Russia

	Sociocultural Adaptation				Psychological Adaptation			
	R^2^	B	SE	β	R^2^	B	SE	β
	.50**				.58**			
Perceived Discrimination		–.12**	.05	–.15**		–.28**	.04	–.43**
Integration Attitude		.39**	.08	.38**		.37**	.06	.45**
Assimilation Attitude		.40**	.08	.34**		.01	.06	.01
Separation Attitude		.01	.08	.01		.15*	.06	.16*
Marginalization Attitude		.02	.06	.02		–.02	.04	–.02
Neuroticism		–.21**	.07	–.20**		–.18**	.05	–.21**
Time in Russia		.03	.04	.06		–.01	.03	–.02
Gender (Female)		–.49**	.18	–.17**		.23	.13	.10
Level of Education		.25*	.13	.14*		.15	.09	.10
Age		–.02	.02	–.06		–.02	.01	–.07

Note. N = 157. **p< .01; *p< .05.

*Table 4* shows the indirect associations of perceived discrimination with adaptation via the person’s acculturation attitudes, after controlling for age, gender, length of time spent in Russia, and neuroticism. We found that perceived discrimination had a significantly negative indirect association via integration attitude with both sociocultural adaptation (β = –.10, *SE* = .04, *LLCI* = –.19, *ULCI* = –.03) and psychological adaptation (β = –.13, *SE* = .04, *LLCI* = –.22, *ULCI* = –.05). None of the other acculturation attitudes were significant partial mediators.

**Table 4:** Indirect Association of Perceived Discrimination with Adaptation

	Sociocultural Adaptation				Psychological Adaptation			
	β	SE	LLCI	ULCI	β	SE	LLCI	ULCI
Integration	–.10	.04	–.19	–.03	–.13	.04	–.22	–.05
Separation	.00	.01	–.03	.03	.03	.02	.00	.08
Assimilation	–.03	.03	–.09	.01	.00	.01	–.02	.02
Marginalization	.01	.02	–.02	.04	.00	.01	–.03	.02

*Note. No. of bootstrap samples for percentile bootstrap confidence interval (CI) = 5000; 95% confidence for all CIs.*

**Figure 2.**
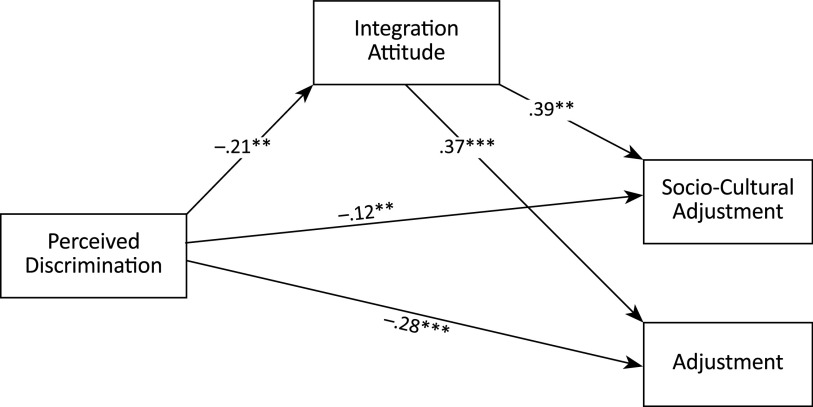
Relationship between Perceived Discrimination, Integration Attitude, and Adaptation

*Figure 2* shows the partial mediation model, depicting integration attitude as the mediator. It had an acceptable fit (χ^2^/df = 4.13; *CFI* = .98; *RMSEA* = .14, *PCLOSE* = .08).

### The Moderating Role of Neuroticism in the Relationship Between Perceived Discrimination, Acculturation Attitudes, and Adaptation

After controlling for the effects of age, gender, education, length of stay in Russia, neuroticism, and perceived discrimination, the results of a series of multiple regression analyses measuring the interaction of perceived discrimination with neuroticism in predicting the acculturation attitudes (see *Table 5*), showed that it was significant in predicting integration attitude (β = -.12, *p* < .05), but not marginalization (β = .07, *p* = .22), assimilation (β = -.02, *p* = .65), or separation (β = .01, *p* = .28).

**Table 5:** Multiple Regression Analysis Predicting Acculturation Attitudes of African Immigrants in Russia

	Integration			Assimilation			Separation			Marginalization		
	ΔR^2^	B	SE	ΔR^2^	B	SE	ΔR^2^	B	SE	ΔR^2^	B	SE
Model Sum.	.21			.18			.07			.14		
Age		.05	.02		.08**	.02		.01	.02		–.01	.03
Gender (female)		–.09	.21		–.31	.19		.11	.20		.21	.27
Time in Russia		.02	.04		–.01	.04		–.03	.04		–.02	.06
Education		–.44*	.15		.01	.13		.21	.14		.18	.19
Perceived Dis-crimination		–.21**	.06		–.07	.05		.15**	.06		.17*	.08
Neuroticism		–.10	.08		.25**	.07		.05	.08		.27**	.11
PD Neuroticism *	.03	–.12*	.05	.00	–.02	.04	.01	–.05	.04	.01	.07	.06

Note. N = 157. **p< .01; *p< .05.

**Figure 3.**
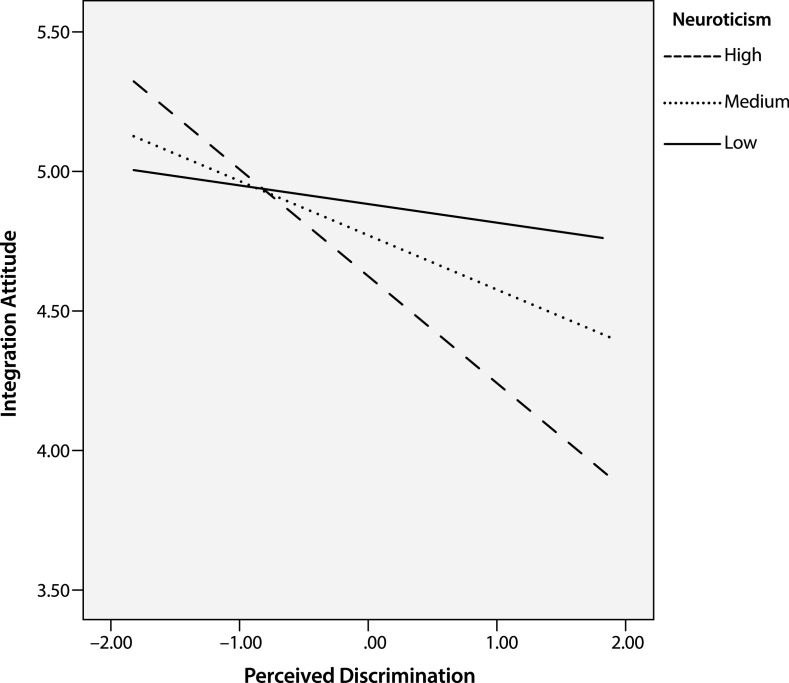
Predicted values of integration attitude, illustrating the interaction between perceived discrimination and neuroticism

*Figure 3* presents the predicted values for the interaction between perceived discrimination and neuroticism in predicting integration attitude.

Comparison of the slopes of the regression lines representing low (16th percentile: *B* = −.06, *SE* = .09, *p* = .47), medium (50th percentile: *B* = −.20, *SE* = .06, *p* < .01), and high neuroticism (84th percentile: *B* = −.38, *SE* = .09, *p* < .001) indicated that the negative association between perceived discrimination and integration attitude was stronger when the participants were higher in neuroticism.

The results of the moderated mediation showed that neuroticism moderated the indirect relationship between perceived discrimination and both sociocultural (*B* = −.05, *SE* = .02, *LLCI* = −.10, *ULCI* = −.01) and psychological adaptation (*B* = −.05, *SE* = .02, *LLCI* = −.09, *ULCI* = −.01), via integration attitude.

Comparison of the indirect effect of perceived discrimination on sociocultural adaptation via integration attitude at low (*B* = −.03, *SE* = .04, *LLCI* = −.11, *ULCI* = .05), medium (*B* = −.08, *SE* = .03, *LLCI* = −.15, *ULCI* = −.03), and high levels of neuroticism (*B* = −.16, *SE* = .05, *LLCI* = −.28, *ULCI* = −.07) indicated that the negative indirect association between perceived discrimination and sociocultural adaptation, via integration attitude was stronger when the participants were higher in neuroticism. The same was the case for psychological adaptation: Low (*B* = −.02, *SE* = .04, *LLCI* = −.10, *ULCI* = .05), medium (*B* = −.08, *SE* = .03, *LLCI* = −.14, *ULCI* = −.02), and high neuroticism (*B* = −.15, *SE* = .05, *LLCI* = −.25, *ULCI* = −.07).

## Discussion

The goal of this study was to determine the moderating role of neuroticism in the relationship between perceived discrimination, acculturation attitudes, and adaptation (sociocultural and psychological) of African immigrants in Russia.

Perceived discrimination has been previously found to predict psychological and sociocultural maladaptation, directly and indirectly through the acculturation attitudes (Giuliani et al., 2018; Te Lindert et al., 2008). Our results supported this claim. We found that perceived discrimination had a direct negative association with both psychological and sociocultural adaptation.

Perceived discrimination may be directly detrimental to psychological well-being in the sense that it can trigger a feeling of rejection (Model et al, 2009). According to social rejection theory, feeling rejected can lead to adverse psychological symptoms, such as low self-esteem, depression, aggression, insecurity, etc. (DeWall & Bushman, 2011; Model et al, 2009). Perceived discrimination may also indirectly predict psychological and sociocultural maladaptation in the sense that it can influence how immigrants/minorities decide to acculturate (*i.e.,* their acculturation attitudes), and this decision can impact their level of adaptation.

According to the existing evidence, immigrants/minorities are less likely to adopt a positive attitude toward integration when they perceive higher levels of discrimination (Berry et al., 2006; Giuliani et al., 2018; Te Lindert et al., 2008). The consequence of this is that their psychological and sociocultural adaptation may suffer, given that integration attitude has repeatedly been demonstrated to positively predict adaptation to varying degrees (Berry et al., 2006; Te Lindert et al., 2008; Bierwiaczonek & Kunst, 2021).

We found support for this assertion. Our results showed that perceived discrimination is negatively associated with integration attitude, which then leads to sociocultural and psychological maladaptation. In other words, the negative associations of perceived discrimination with both psychological and sociocultural adaptation were partially mediated by integration attitude.

In essence, as the levels of perceived discrimination of African immigrants in Russia increased, the immigrants increasingly veered away from adopting an integration attitude (*i.e*., not inclined to preserving their heritage culture and participating in the mainstream culture simultaneously): this led to them to be less adapted psychologically and socioculturally. Perceived discrimination may negatively predict integration attitude in the sense that perceived discrimination is seen as a threat to a group’s esteem/value. According to the theory of group esteem threat, when people feel that the value of their in-group is threatened (*e.g*., via discrimination), they may try to dis-identify from it in order to protect their self-esteem, while simultaneously becoming increasingly averse toward the source of the threat (*e.g.,* the discriminatory host society for immigrants/minorities) (Tajfel & Turner, 1986).

Based on the nature-nurture paradigm in psychology, we understand that dispositional factors interact with social/environmental factors to determine a certain outcome. Thus, when dealing with humans, we can expect individual differences in reactions to similar sociocultural conditions due to differences in personality characteristics. With the knowledge that perceived discrimination is an acculturative stress-or that elicits negative emotions and psychological distress (Berry, 2006; DeWall & Bushman, 2011; Model et al, 2009; Stein et al, 2016), we wanted to see whether neuroticism interacts with perceived discrimination to influence acculturation attitudes, and how this might affect adaptation. In other words, does the level of neuroticism of African immigrants in Russia influence how they react to perceived discrimination in terms of how they decide to acculturate (acculturation attitude) under these conditions? And does this consequently impact their level of adaptation?

Emotional reactivity, a component of neuroticism, is known to intensify the direct positive relationship between perceived discrimination and depressive symptoms (Stein et al., 2016). However, there is still a gap in knowledge about how neuroticism interacts with perceived discrimination to predict the acculturation attitudes, which indirectly impact adaptation. This study sought to fill this gap by investigating the moderating role of neuroticism in the indirect relationship between perceived discrimination and adaptation via the acculturation attitudes of African immigrants in Russia.

The results showed that neuroticism strengthened the negative relationship between perceived discrimination and integration attitude, thereby indirectly strengthening its detrimental effect on adaptation (both psychological and sociocultural). At higher levels of perceived discrimination, African immigrants higher in neuroticism were significantly less likely to adopt an integration attitude compared to those lower in neuroticism. Thus, even despite perceiving similarly high levels of discrimination, those higher in neuroticism were significantly more maladapted psychologically and socioculturally than those lower in neuroticism.

Neuroticism is characterized by a higher proclivity for psychological distress caused by negative emotions and stress (Gunthert et al., 1999; McCrae & John, 1992; Steenhaut et al., 2018), as well as an increased sensitivity to rejection. (Brookings et al., 2003; Wilhelm et al., 2004). Given that perceived discrimination is a form of acculturative stress (Berry, 2006) and a form of rejection that elicits negative emotions and causes psychological distress (*e.g*., low self-esteem, depression, aggression, insecurity, and so on), it’s highly probable that African immigrants higher in neuroticism were more affected by the negative emotions and psychological distress elicited by perceiving higher levels of discrimination. And since they also tend to be relatively higher in sensitivity to rejection, they had a stronger reaction, veering away from adopting an integration attitude at a significantly higher rate than those lower in neuroticism. As a consequence of this, those higher in neuroticism were less psychologically and socio-culturally adapted to life in Russia.

## Conclusion

In their research on sociocultural adaptation of Africans in Russia, Bondarenko et al. (2009) conducted interviews with some African immigrants on their experiences of living in Russia. They found that most of them, including those who had a relatively good command of the Russian language, shared experiences of discrimination and appeared to be psychologically and socio-culturally maladapted to the Russian society. However, there are reports of cases where, despite being under similar conditions of perceived discrimination, some African immigrants were relatively well adapted in Russian society (Simmons, 2014). Thus, in this study, we sought to investigate whether the personality trait of neuroticism could somewhat account for this variation by moderating the relationship between perceived discrimination, acculturation attitudes, and adaptation.

The results of our analysis indicated that neuroticism was a significant moderator in the relationship between perceived discrimination, integration attitude, and adaptation. Specifically, we found that, compared to neurotic African immigrants, those who were emotionally stable did not significantly veer away from adopting an integration attitude, even under similarly high levels of perceived discrimination. This consequently led to them being less impacted by the indirect negative effects of perceived discrimination on their psychological and sociocultural adaptation. Considering that neuroticism makes one more prone to experiencing negative emotions and psychological distress (Costa & McCrae, 1985), we extrapolated that the African immigrants higher in neuroticism might have been more impacted by the negative emotions and psychological distress elicited by perceived discrimination. This could explain why they had a stronger negative reaction toward integration.

Future studies could investigate how different personality traits could interact with each other to moderate the relationship between perceived discrimination and acculturation orientations. For example, considering that extraverts are in their comfort zone when being outgoing, sociable, and gregarious, they may not be as likely as introverts are to withdraw from participation in the mainstream culture, even under conditions of perceived discrimination. In this sense, an extrovert with a high level of neuroticism may find it even more difficult to disengage from involvement with the mainstream culture, as his/her neuroticism might exacerbate the distress under conditions of social withdrawal. This could indicate that extraversion would mitigate neuroticism’s moderating influence in the relationship between perceived discrimination and acculturation orientations.

## Limitations

It is important to note some of the limitations of this study. First, due to its cross-sectional design, we cannot conclude causality. Secondly, the sample size was only suitable (at a power of .8) for detecting moderate effects in regression. Thus, there is a risk of a type 2 error since significant smaller effects may have been missed.

Despite some of the limitations of this study, its findings provide a plausible partial explanation as to why some African immigrants in Russia, under similar conditions of high perceived discrimination, were relatively well-integrated and adapted compared to others. This may have been a result of their lower levels of neuroticism.

## Appendix

### Questionnaire items

*Note:* All answers except those on socio-demographics are measured with a 7-point Likert scale (1 = *Strongly Disagree*; 7 = *Strongly Agree*); ® Reversed item.

#### Socio-demographics

1. Age (input)
2. Gender (male, female, other)
3. Country of Origin (input)
4. Level of Education (no education, primary, secondary/high school, bachelors, masters, doctorate)
5. Length of Time in Russia (< 6 mths, 6 mths – 1 yr., 1-1.5yrs, 1.5-2 yrs. …… >4 yrs.)

#### Neuroticism

Instruction*: Indicate your level of agreement with the following about yourself currently, not how you wish to be in the future.*

- I seldom feel blue.
- Most of the time I am relaxed; I handle stress well. ®
- I am easily upset.
- I often feel anxious about what could go wrong.
- I have frequent mood swings.

#### Perceived Discrimination Scale

Instruction*: Indicate your level of agreement with the following about your experience in Russia.*

1. I feel as if I am ignored or excluded from opportunities such as good jobs in Russia because I am African.
2. In general, I feel as if Russians view Africans in a negative way and act unfairly towards us.
3. I feel as if I am not wanted, accepted, or welcomed in Russia because I am African.
4. I don’t quite feel valued or respected by my Russian colleagues at school or work because I am African.

#### Acculturation attitude

Instruction*: Indicate your level of agreement with the following about your experience in Russia.*

1. Friendship domain.
  - *Assimilation*: I find myself a lot more inclined toward having Russian friends than I am toward having African friends.
  - *Separation*: I find myself a lot more inclined toward having African friends than I am toward having Russian friends.
  - *Integration*: I find that I have a balanced inclination toward having Russian and African friends.
  - *Marginalization*: I don’t really have an inclination toward having either Russian or African friends. These days, it’s hard to find someone I can really relate to.
2. Society domain.
  - *Assimilation:* Encouraging Africans to stick together and unequivocally practice our culture in Russia only hinders our acceptance into Russian society.
  - *Separation*: Because we live in Russia, we are losing our heritage to the Russian culture. Thus, I decide to emphasize my distinct African identity and minimize my participation in the Russian culture.
  - *Integration*: While living in Russia, I strive to retain my African cultural heritage and lifestyle, while also participating fully in various aspects of Russian culture.
  - *Marginalization*: I don’t really care about preserving my African cultural heritage nor participating in the Russian culture.
3. Events domain.
  - *Assimilation*: I tend to value attending Russian events like parties, ceremonies, etc. significantly more than I value attending African events in Russia.
  - *Separation*: I value attending African events in Russia significantly more than I value Russian events.
  - *Integration*: I value attending African events in Russia as well as Russian events more or less equally.
  - *Marginalization*: Neither African events nor Russian events hold much value for me. I just don’t care very much about either of them.
4. Lifestyle domain.
  - *Assimilation*: I have more of a Russian lifestyle than my African cultural heritage lifestyle now that I am living in Russia.
  - *Separation*: My lifestyle is nothing like that of the Russians. I try to maintain my African cultural lifestyle as much as I can.
  - *Integration*: My lifestyle in Russia is a substantial blend between my African cultural heritage and the Russian lifestyle.
  - *Marginalization*: I don’t live according to either my African cultural heritage or the Russian lifestyle. I live in my unique way.

#### Sociocultural adaptation scale

1. I find it easy to make Russian friends.
2. I get along easily with Russians.
3. I have a relatively good understanding of the social norms in Russia, *e.g*., what is appropriate to say, how to act, dress, etc.
4. I find it easy to make myself understood by and to understand Russian people.

#### Psychological adaptation scale

1. I am happy with my day-to-day life in Russia.
2. I feel out of place, as if I don’t fit in, living in Russia. ®
3. I feel uncomfortable being around Russians. ®
4. I feel a sense of freedom living in Russia.
